# Label-Free Proteomic Approach to Study the Non-lethal Effects of Silver Nanoparticles on a Gut Bacterium

**DOI:** 10.3389/fmicb.2019.02709

**Published:** 2019-12-04

**Authors:** Guido Domingo, Federica Villa, Candida Vannini, Elisa Garuglieri, Elisabetta Onelli, Marcella Bracale, Francesca Cappitelli

**Affiliations:** ^1^Department of Biotechnology and Life Sciences, Università degli Studi dell’Insubria, Varese, Italy; ^2^Department of Food, Environmental and Nutritional Sciences, Università degli Studi di Milano, Milan, Italy; ^3^Department of Biosciences, Università degli Studi di Milano, Milan, Italy

**Keywords:** label-free proteomics, silver nanoparticles, non-lethal effects, gut biofilm, acute, chronic exposure

## Abstract

Among all the food-related nanoparticles consumed every day, silver nanoparticles (AgNPs) have become one of the most commonly utilized because of their antimicrobial properties. Despite their common use, the effects of sublethal concentrations of AgNPs, especially on gut biofilms, have been poorly investigated. To address this issue, we investigated *in vitro* the proteomic response of a monospecies *Escherichia coli* gut biofilm to chronic and acute exposures in sublethal concentrations of AgNPs. We used a new gel- and label-free proteomic approach based on shotgun nanoflow liquid chromatography–tandem mass spectrometry. This approach allows a quantification of the whole proteome at a dynamic range that is higher than the traditional proteomic investigation. To assess all different possible exposure scenarios, we compared the biofilm proteome of four treatments: (i) untreated cells for the control treatment, (ii) cells treated with 1 μg/ml AgNPs for 24 h for the acute treatment, (iii) cells grown with 1 μg/ml AgNPs for 96 h for the chronic treatment, and (iv) cells grown in the presence of 1 μg/ml AgNPs for 72 h and then further treated for 24 h with 10 μg/ml AgNPs for the chronic + acute treatment. Among the 1,917 proteins identified, 212 were significantly differentially expressed proteins. Several pathways were altered including biofilm formation, bacterial adhesion, stress response to reactive oxygen species, and glucose utilization.

## Introduction

Nanoparticles (NPs) have unique sizes, shapes, and structures, and this makes them ideal components for various applications (Carbone et al., 2015). Silver nanoparticles (AgNPs), in particular, are exponentially used in medical, health, industrial, and precision agricultural sectors because of their unique features, such as chemical, optical, electrical, and thermal properties (Zhang et al., 2016; Duhan et al., 2017). Moreover, AgNPs are usually added to the polymeric matrices of food packaging to enhance food protection to extend its shelf-life. However, AgNPs may transfer and accumulate through the food chain, risking the human health (Fröhlich and Fröhlich, 2016; Tsiaoussis et al., 2019). The effects of AgNPs on the intestinal human microbiota, the so far neglected “essential organ” (O’Hara and Shanahan, 2006), should be considered. The microbiota provides several advantages to the host through a variety of physiological functions: host immunity promotion, pathogens control, and energy harvest (Thursby and Juge, 2017). Alterations in the intestinal microbiota have been associated with many different diseases, ranging from metabolic syndrome to diabetes atherosclerosis, to colorectal cancer (Yang and Kweon, 2016).

In the human gastrointestinal microbiota, *Escherichia coli* can commonly exist as a commensal, a probiotic, or a pathogenic bacterium (Rossi et al., 2018). To enlighten the effect of ingested AgNPs on intestinal bacterial biofilm and human intestinal epithelial cells, we recently analyzed the physiological response of a model system composed of *E. coli* monospecies biofilm and Caco-2 cells exposed to 1 μg/ml of AgNPs (Garuglieri et al., 2018). Previously, it was claimed that a AgNP concentration of 1 μg/ml is representative of actual food-related ingestion (Gottschalk et al., 2013; Kuorwel et al., 2015; Fröhlich and Fröhlich, 2016). In Garuglieri et al. (2018), it was demonstrated that (1) bacterial biofilm plays an important role in protecting intestinal epithelial cells from AgNPs genotoxic effects, and (2) under anaerobic conditions, AgNPs seriously impact the *E. coli* biofilm.

This study aimed to explore the proteomic changes that occur when *E. coli* biofilm is exposed to AgNPs. To this end, *E. coli* biofilm was cultured using Transwell setup as described in Garuglieri et al. (2018). AgNP exposure was performed to simulate the chronic and/or the acute effects of AgNPs on mature bacterial biofilms.

To investigate the impact of silver nanoparticles at realistic concentrations, we evaluated the chronic and the acute effects of sublethal AgNP doses at 1 and 10 μg/ml. We used a new proteomic approach based on shotgun nanoflow liquid chromatography–tandem mass spectrometry (LC-MS/MS) to obtain a global view of *E. coli* biofilm responses to the different AgNP exposures. We have thus highlighted protein changes triggered by AgNPs and identified common responses among the treatments as well as specific alterations.

## Materials and Methods

### Silver Nanoparticles

The AgNPs were purchased from BioPure, NanoComposix (San Diego, CA, United States). According to the supplier, their hydrodynamic diameter is <20 nm, and their negative zeta potential is −27.3 mV (Malvern Zetasizer Nano ZS). AgNP size was assessed to be 14 ± 0.3 nm using transmission electron microscopy (TEM) by our team’s previous work (Garuglieri et al., 2016). AgNPs were stored at 4°C and, just before use, diluted to 1 or 10 μg/ml in bidistilled water or in Tryptic soy broth medium (Conda, Italy).

### Experimental Setup and Bacterial Biofilm Exposure to AgNPs

*Escherichia coli* MG 1655 biofilms were grown under anaerobic conditions on polycarbonate membranes (PC, Whatman Nucleopore, 2.5 cm diamater, 0.2 μm pore diameter) in Transwell systems (Greiner bio-one) as described by Garuglieri et al. (2018). Briefly, polycarbonate membranes inoculated with 5 × 10^6^ cells were placed in the Transwell inserts. One milliliter of prereduced Tryptic soy broth medium with and one without 1 μg/ml AgNPs were added to the plate wells (basolateral medium). As 1 μg/ml represented the realistic human intake dosage, both the acute and chronic treatments were evaluated at this concentration. Three different treatments were assayed: (1) AgNPs added for 24 h directly onto the surface of an established biofilm, which was pregrown for 72 h without AgNPs (acute treatment, effect on established biofilm); (2) biofilm grown for 96 h with the addition of 1 μg/ml AgNPs dispersed in the basolateral medium (chronic treatment, effect on biofilm formation); (3) biofilm grown for 72 h with the addition of 1 μg/ml AgNPs dispersed in the basolateral medium and then further treated for 24 h with 10 μg/ml AgNPs added directly onto the surface of the established biofilm (chronic + acute treatment, effect of a short and aggressive AgNPs exposure to an adapted biofilm).

The controls for all treatments were defined as pristine biofilms grown for 96 h. Experiments were conducted in triplicate. The growth profile and reactive oxygen species (ROS) generation of *E. coli* under condition 1 (acute treatment) and condition 2 (chronic treatment) compared to control samples can be found in Garuglieri et al. (2018).

### Liquid Chromatography–Mass Spectrometry Analysis

After treatments, polycarbonate membranes with adhering biofilm were removed from the Transwell setups, transferred to tubes containing 1 ml of sterile phosphate-buffered saline solution (Medicago AB, Uppsala, Sweden), vortexed for 1 min, and sonicated for 3 min in a water bath (Sonica Ultrasonic Cleaner, Soltec, Milan, Italy). The cell pellet was resuspended in 4% (*w*/*v*) sodium dodecyl sulfate, 100 mM Tris–HCl pH 7.6 and 100 mM dithiothreitol, boiled for 5 min and centrifuged for 30 min at 13,000 rpm (Ölander et al., 2016). Proteins were quantified with 2-D Quant Kit (GE healthcare); 200 μg of protein was then trypsinized using the two-digestion-filter-aided sample preparation method (Wiśniewski and Mann, 2012). Digested samples (five replicates for each treatment) were directly analyzed on a QExactive mass spectrometer coupled to a nano EasyLC 1000 (Thermo Fisher Scientific Inc., Waltham, MA, United States). The solvent composition for channel A was 0.1% formic acid, while for channel B, it was 0.1% formic acid and 99.9% acetonitrile. For each sample, 4 μl of peptides was loaded on a self-made column (diameter × length: 75 μm × 150 mm) packed with reverse-phase C18 material (ReproSil-Pur 120 C18-AQ, 1.9 μm; Dr. Maisch GmbH, Ammerbuch, Germany) and eluted at a flowrate of 300 nl/min with a gradient from 2 to 35% B in 80 min, from 35 to 47% B in 4 min, and from 47 to 98% B also in 4 min. Samples were acquired randomly. The mass spectrometer was operated in data-dependent mode, acquiring full-scan MS spectra (300–1,700 *m*/*z*) at a resolution of 70,000 at 200 *m*/*z* after accumulation to a target value of 3,000,000, followed by higher-energy collision dissociation fragmentation on the 12 most intense signals per cycle. Higher-energy collision dissociation spectra were acquired at a resolution of 35,000 using a normalized collision energy of 25 and a maximum injection time of 120 ms. The automatic gain control was set to 50,000 ions. Charge state screening was enabled, and singly and unassigned charge states were rejected. Only precursors with an intensity above 8,300 were selected for MS/MS (2% underfill ratio). Precursor masses previously selected for MS/MS measurement were excluded from further selection for 30 s, and the exclusion window was set at 10 ppm. The samples were acquired using internal lock mass calibration on *m*/*z* 371.1010 and 445.1200.

### LC-MS/MS Data Analysis

Raw data obtained from the mass spectrometer were processed with MaxQuant v. 1.5.3.3^1^ for peptide identification and quantifications. Identification was performed with MaxQuant integrated Andromeda search engine against the UniProt *E. coli* protein database (1,352,175 entries), downloaded September 29, 2016. The search criteria were the following: two missed cleavages, fixed modification of cysteine (carbamidomethylation), variable modifications of methionine (oxidation) and phosphorylation on serine, threonine, and tyrosine, and minimum peptide length of six amino acids. The minimum criteria for protein identification were two peptides and one unique peptide and precursor mass tolerance of 4.5 ppm for the main search. Label-free quantification, the match between runs (time window of 0.7 min), and the target-decoy search strategy (revert mode) options were enabled. Identifications were accepted with a false discovery rate (FDR) of 1% for peptide and proteins. The mass spectrometry proteomics data have been deposited in the ProteomeXchange Consortium via the PRIDE (Perez-Riverol et al., 2019) partner repository under the dataset identifier PXD014096.

### Proteomics Data Processing

Once identified, all protein groups without contaminants and decoy entries as well as groups without “only identified by site” were chosen. Resulting protein groups were considered as identified when values were detected in at least two biological replicates and in one analytical group. This process led to missing values for one or the other condition. Missing values were estimated from the dataset by an in-house tool, based on two criteria for each sample, depending on whether one or more missing values were observed for each entry. When two values were available, the imputation of the missing value was set to a random value within an interval of one-fourth of the entire sample standard deviation centered on the entry average. While only one or no value was available, the imputation of the missing value was set to a random value within an interval of one-fourth of the standard deviation of all sample values centered on the global minimum value of all samples in the dataset.

### Statistical Analysis

To determine the differentially expressed proteins (DEPs) among considered conditions, we performed a multiple sample test using analysis of variance (ANOVA) with a permutation-based FDR cutoff of 0.05. Statistical analysis was performed on each dataset (means ± SD, *n* = 4) with custom *R* scripts. One-way ANOVA and *post hoc* Tukey’s test were done using two R statistical packages, limma, and cp4p^2^.

### Principal Component Analysis and Hierarchical Clustering

To assess the quality of our datasets, Perseus software (MaxQuant, v1.11, Martinsried, Germany) was used for PCA. We also built a Venn diagram by submitting to VENNY^3^ the entire dataset of identified and quantified proteins.

Hierarchical clustering analysis was performed using Perseus with the following settings: Row, Column distance calculated using the Euclidean algorithm; Row, Column linkage Complete.

Hierarchical clustering heat map of significantly changing proteins was performed using the *Z*-score calculation on log2 intensity values.

### EcoCyc Analyses

For all differentially abundant proteins, present in each selected cluster, a Blastp was performed against ecoli.fsa database (4,287 sequences, 1,342,714 total letters) to obtain EcoCyc accession IDs from Uniprot protein IDs.

EcoCyc is a bioinformatics database available at EcoCyc.org that describes the genome and the biochemical machinery of *E. coli* K-12 MG 1655.

Best EcoCyc accession protein IDs were used for omics data analysis in EcoCyc. Paint multiomics data onto a Pathway Collage options were used to generate a user-customizable diagram containing a set of pathways of interest, overlaid with multiomics data.

### Transmission Electron Microscopy Observations

Cells were fixed in cold 0.05 M Hepes buffer, pH 7.4, containing 2% glutaraldehyde and 2% formaldehyde added in Transwell systems. To prevent biofilm disorganization during following TEM embedding procedure, the membranes with biofilm were carefully cut with a razor blade, and these pieces were laid in a drop of 3% sodium alginate (in 0.05 M Hepes buffer, pH 7.4) and immediately solidified with CaCl_2_ 0.2 M. The samples were than repeatedly rinsed in the same Hepes buffer, postfixed in 1% osmium tetroxide, for 1 h at 4°C, dehydrated in ethanol, and embedded in London White resin. Ultrathin sections, obtained using a Reichert Jung Ultracut E microtome, were stained with 3% uranyl-acetate and lead citrate. Observations were performed with an EFTEM LEO 912AB transmission electron microscope (Zeiss, Jena, Germany) operating at 80 kV.

## Results and Discussion

### *E. coli* Biofilm Proteome Under AgNPs Treatments

To gain insights into the response of *E. coli* to AgNPs, we compared the total biofilm proteome of four different samples: untreated biofilm (control), biofilm treated with AgNPs for 24 h (acute treatment), biofilm grown in the presence of AgNPs for 96 h (chronic treatment), and biofilm grown in the presence of AgNPs for 72 h and retreated with a higher dose of AgNPs for 24 h (chronic + acute treatment). Among the 1,917 proteins identified, 212 (11.26%) were significantly differentially expressed proteins (ANOVA test, FDR of 5%) (Supplementary Table S1).

### Principal Component Analysis and Hierarchical Clustering

**FIGURE 1:**
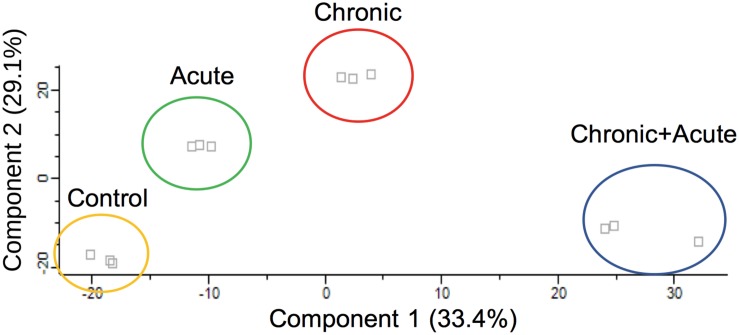
Principal component analysis (PCA) score plot of total proteome data. Samples with different treatments (control, acute, chronic, chronic + acute) were indicated by colors. *X*-axis and *Y*-axis were labeled with the first principal component and the second principal component, accounting for 33.4 and 29.1% total variation, respectively.

**FIGURE 2:**
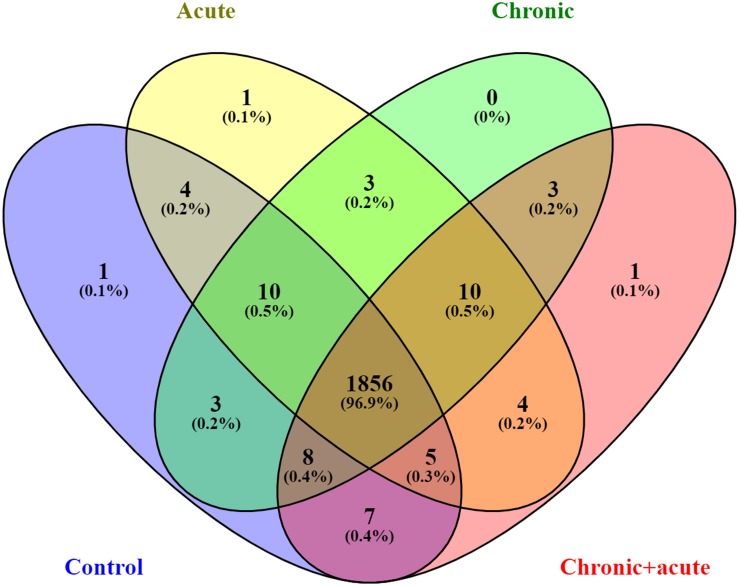
Venn diagrams of the identified and quantified proteins in the four treatments.

Principal component analysis of the identified proteins across all samples revealed that the proteome of biofilms under different AgNP exposure assembled separately from each other into distinctly isolated clusters (Figure 1). The Venn diagram in Figure 2 compared the number of expressed proteins across all treatments, showing common and unique proteins among the different exposure conditions. In particular, the Venn diagram showed 1,856 proteins (96.9%) commonly regulated in all the treatments.

**FIGURE 3:**
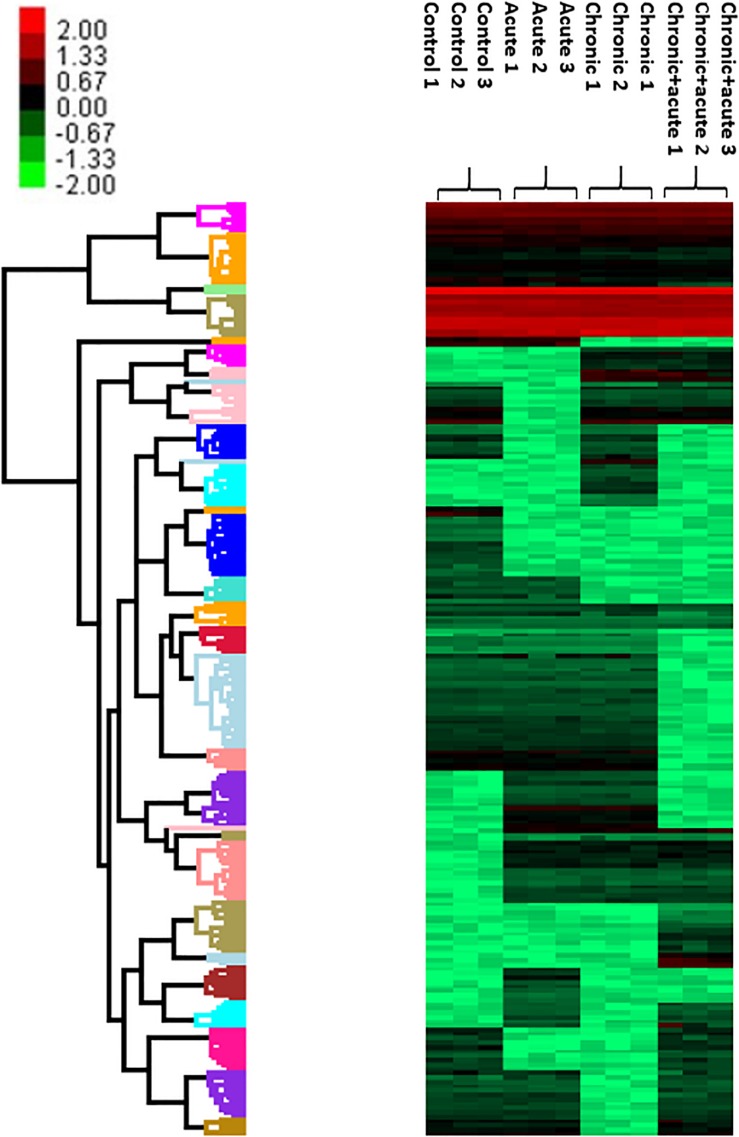
Heatmap and hierarchical clustering of 212 differentially expressed proteins (DEPs) with false discovery rate (FDR) of 5%. Colors ranging from green to red represent protein abundance from the highest level of downregulation to the highest level of upregulation, respectively.

**FIGURE 4:**
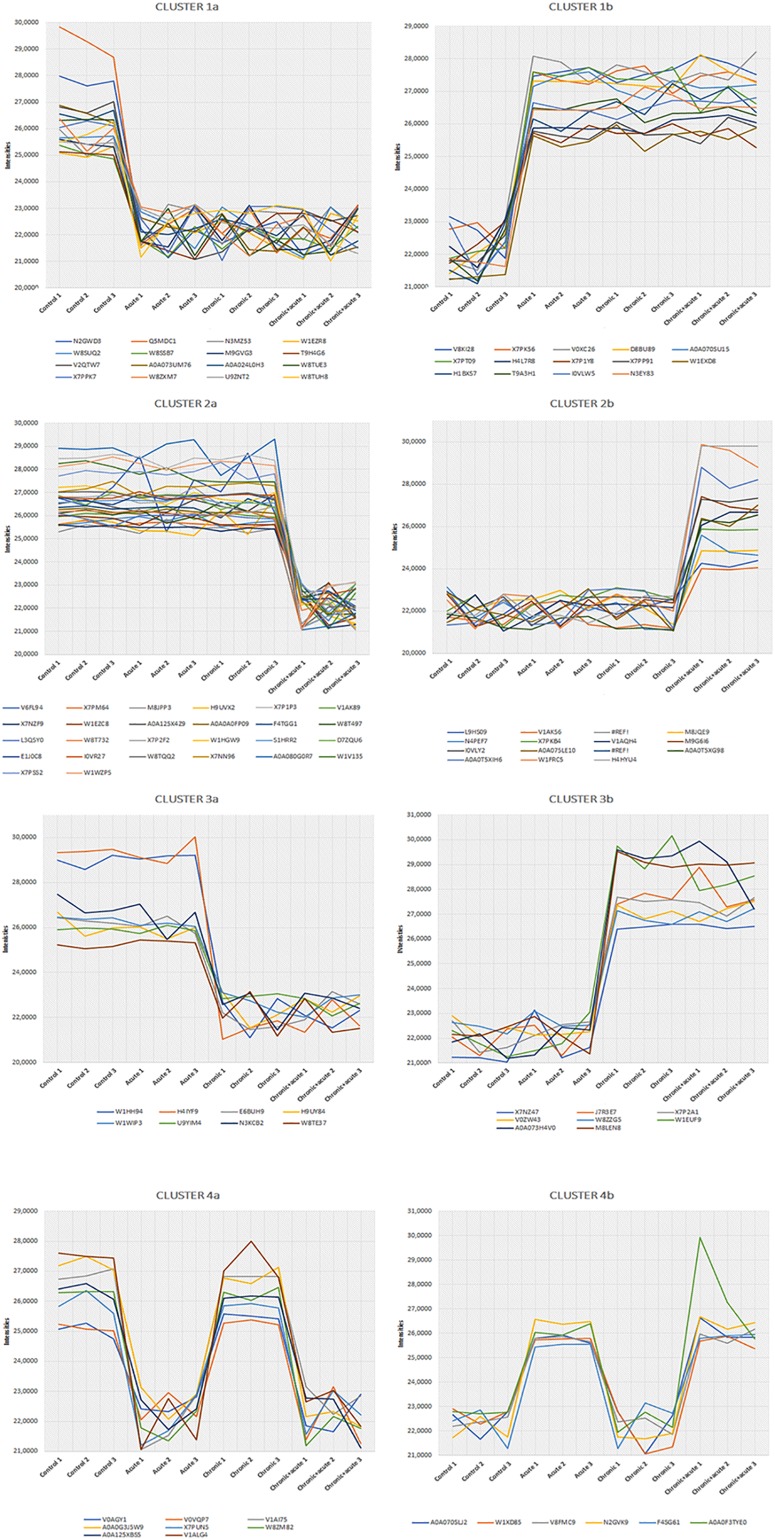
Eight most interesting and prominent expression profiles found in cluster analysis. Differentially expressed proteins (DEPs) belonging to each profile are represented by colored lines.

We also performed a hierarchical clustering analysis and produced a heatmap to identify groups of proteins that display similar expression patterns across all treatments. The hierarchical clustering of the 212 significantly differentially expressed proteins revealed distinct expression patterns (Figure 3). Heat map showed the clustered data, where each colored cell represents the protein abundance value. Column-wise clustering of triplicate measurements for each treatment demonstrated that biological variation was greater in the most severe experimental AgNP treatment considered (chronic + acute treatment) with respect to the control. Row-wise clustering revealed different protein groups based on the modulation of their expression. Eight expression clusters of interest with their relative intensity values are shown in Figure 4.

Clusters 1a and 1b were composed of proteins with increasing and decreasing abundances after all AgNP treatments, respectively. Clusters 2a and 2b included proteins that decrease and increase only under the chronic + acute AgNP treatment, respectively. Clusters 3a and 3b showed proteins after chronic treatments with decreasing and increasing abundances, respectively. Clusters 4a and 4b represented proteins with low or high abundance after acute AgNP treatment, respectively.

Table 1 summarizes the observed main effects and the corresponding proteins. Supplementary Table S2 lists all proteins belonging to the eight expression clusters and their respective localizations, intensity values, and log2 fold changes (log2FC). Minimum log2FC considered in this study were −2.56 and 2.21, which are very high values (Supplementary Table S2).

**TABLE 1:** Main effects observed and, for each type of treatment, the proteins related.

**Cluster n.**	**Type of treatments**	**Biofilm alterations**	**Cell wall/membrane alterations**	**Response to stress**	**DNA damage**	**Glucose utilization**
Clusters 1a–1b	All AgNPs treatments	↓ 2-methylcitrate synthase (N3MZ53; log2FC = −2.98, −3.06, −3.21) ↓ GTP cyclohydrolase II (W8SSB7; log2FC = −3.32, −3.21, −3.21) ↑Glyoxalase I (T9A3H1; log2FC = 4.57, 4.44, 4.43)	↓ ABC transporter-binding protein MlaB (N2GWD3; log2FC = -5.95, −5.68, −5.84) ↑ 1-Acylglycerol-3-phosphate *O*-acyltransferase (X7PK56; log2FC = 4.74, 4.80, 4.82)	(↓ GTP cyclohydrolase II (W8SSB7; log2FC = −3.21, 3.21, 3.21) ↓ TusD subunit of the sulfur transfer protein complex (W8TUH8; log2FC = −3.68, 2.90, 3.60)	–	↓ Malate dehydrogenase (Q5MDC1; log2FC = −6,93, 7.14, 6.87) (Sugar phosphatase (X7PPK7; log2FC = −3.82, −3.75, −3.93)
Clusters 2a–2b	Only chronic + acute AgNPs treatments	↓ Fimbrial proteins (D7ZQU6; log2FC = 0.07, −0.38, −4.24) ↓ Beta-glucuronidase (F4TGG1; log2FC = −0.01, −0.02, −3.98) ↑ UTP-glucose-1-phosphate uridylyltransferase (W1FRC5; log2FC = −0.14, 0.24, 7.26) ↑ Disulfide bond reductase (H4HYU4; log2FC = −0.51, 0.27, 7.64)	↓ L,D-transpeptidase LdtD (A0A125X4Z9; log2FC = −0.20, −0.03, −4.18) ↓ Bacteriophage N4 receptor (L3QSY0; log2FCtransporter (W8TQQ2; log2FC = −0.21, −0.14, −5.26) (Phosphate acyltransferase (W1HGW9; log2FC = −0.44, −0.43, −5.28)	↓ Potassium efflux system KefA (V1AK89; log2FC = 0.11, 0.01, −4.01) (↓ PutA protein (V6FL94; log2FC = 0.34, 0.50, −4.28) ↑ Multiple antibiotic resistance regulators MarR (V1AQH4; log2FC = 0.33, 0.42, 4.65)	–	↑ Sugar transport-related regulator SgrR (V1AK56; log2FC = 0.51, −0.29, 2.46)
Clusters 3a–3b	Only chronic treatments	↓ C-di-GMP phosphodiesterase YhjK (H9UY84; log2FC = −0.26, −3.83, −3.41) (↑ Cell division activator (CedA) (W8TE37; log2FC = 0.24, −3.05, −3.25) ↑ Biofilm regulator protein bssR (W1EUF9; log2FC = 0.33, 7.80, 6.45)	–	–	↑ Formamidopyrimidine-DNA glycosylase (X7NZ47; FC = 0.33, 7.80, 6.45)	–
Clusters 4a–4b	Only acute treatments	↓ Flagellar brake protein YcgR (V0AGY1; log2FC = −2.51, 0.47, −2.90) ↓ Tetratricopeptide repeat (TPR) region (V1ALG4; log2FC = −5.79, −0.25, −5.01) ↑ Sensor kinase protein RcsC (F4SG61; log2FC = 3.34, 0.21, 3.70) ↑ Elongation factor Tu (A0A0F3TYE0; FC = 1.15, 0.98, 1.21) ↑ Permease-cytosine transporter (A0A070SLJ2; log2FC = 3.36, −0.47, 4.90) ↑ Dipeptide transport system permease protein dppB (W1XD85; log2FC = 3.10, −0.93, 2.98)	↓ Tetratricopeptide repeat (TPR) region (V1ALG4; log2FC = −5.79, −0.25, −5.01)	–	↓ Primosomal protein (X7PUN5; log2FC = −4.00, −0.78, −3.66)	–

*For each protein entry name (www.uniprot.org) and log2 fold changes (log2FC) (respectively: chronic treatment vs. control, acute treatment vs. control, chronic + acute treatment vs. control) are reported.*

#### Clusters 1a and 1b

Clusters 1a and 1b consisted of 16 and 14 proteins, respectively, the abundances of which decreased and increased under AgNP exposure, regardless of the nature of treatment.

In cluster 1a, we found downregulation of proteins related to biofilm formation that confirmed the biofilm alterations caused by AgNPs reported by Garuglieri et al. (2018). In particular, the protein MlaB, a part of the ABC transporter complex MlaFEDB, is a small cytoplasmic protein that is very important for both the proper assembly and activity of the ABC complex (Thong et al., 2016). When ABC complex is altered, the plasma membrane shows defects in phospholipid asymmetry and slow rates of trafficking of other membrane components, especially the phosphatidylcholine (Gulshan and Moye-Rowley, 2011). Phosphatidylcholine has been found in many bacteria for its influence on motility and biofilm formation (Klüsener et al., 2009). In all AgNP treatments, we also observed the downregulation of 2-methylcitrate synthase and protein guanosine-5′-triphosphate (GTP) cyclohydrolase II, both of which have been shown to be involved in biofilm formation (Schembri et al., 2003; Mhatre et al., 2016).

The GTP cyclohydrolase II in *E. coli* is regulated by SoxRS system, the major pathway for superoxide stress response (Koh et al., 1996). This finding suggested that exposure to AgNPs increased oxidative stress in biofilms. In fact, it has been demonstrated that *ribA*, the gene encoding GTP cyclohydrolase II, is involved in the removal of 8-oxo-dGTP, which is a toxic product generated by ROS-mediated oxidation of the guanine nucleotide pool (Zhao and Drlica, 2014). GTP cyclohydrolase II protein also catalyzes the initial steps of the riboflavin biosynthesis pathway. Riboflavin (vitamin B_2_) is the direct precursor of the flavin cofactors flavin mononucleotide and flavin adenine dinucleotide. Flavin mononucleotide and flavin adenine dinucleotide accept electrons during oxidation of various compounds, such as amino acids, carbohydrates, and fatty acids. These cofactors are also required in redox reactions during DNA repair (Yurgel et al., 2014).

TusD subunit of the sulfur transfer protein complex belonged to cluster 1a. Introducing thiomodifications to transfer ribonucleic acid (tRNA), the TusD subunit ensures a higher stability of tRNA, enhances the translation efficiency, and prevents the frameshifting during translation (El Yacoubi et al., 2012). In addition, the end turnover of tRNA in *E. coli* is reported as one of the first responses to stress (Montero et al., 2009; Villa et al., 2012).

In the biofilm microbiota, the gene expression is characterized by high abundance of transcripts, where most of them are related to glucose utilization, peptide degradation, and amino acid transport (Schwab et al., 2014). We found a reduction in the abundance of key enzymes of glucose and glucose-1-phosphate degradation pathways, such as the malate dehydrogenase and the sugar phosphatase (Wildberger et al., 2015). These findings suggested an alteration of the metabolic activity. In the tricarboxylic acid cycle, malate dehydrogenase catalyzes the reversible oxidation of malate to generate oxaloacetate using nicotine adenine dinucleotide (NAD+) as an electron acceptor (Sutherland and McAlister-Henn, 1985). Decreased gluconeogenic activity might indicate a decreased requirement for new amino sugars for cell wall synthesis. This observation corresponded with the decreased expression of genes involved in cell wall synthesis and cell division as found by Clark et al. (2012).

Cluster 1b grouped all upregulated proteins in all AgNP treatments. In this cluster, we observed an upregulation of the glyoxalase I. Studies by Santi et al. (2014) and Subhadra et al. (2019) reported that this enzyme is upregulated in biofilms, and it can also act in the detoxification of methylglyoxal (MG), which is a side product of several metabolic pathways. The toxicity of MG is believed to be attributed to its ability to interact with the nucleophilic centers of macromolecules, such as deoxyribonucleic acid (DNA) (Ferguson et al., 1998). In bacteria like *E. coli*, the glutathione-dependent glyoxalase I–II pathway is the primary route for MG detoxification. During this detoxification process, the glutathione is restored because it is an important cellular antioxidant often used for biofilm protection (Santi et al., 2014).

The observed upregulation of the protein 1-acylglycerol-3-phosphate *O*-acyltransferase indicated an activation of the phospholipid biosynthesis pathway through the cytidine diphosphate-diacylglycerol intermediate. In this pathway, the 1,2-diacyl-*sn*-glycerol-3-phosphate is converted to its activated form, the cytidine diphosphate-diacylglycerol, which is an important intermediate in the biosynthesis of all membrane glycerophospholipids (Swartley et al., 1995).

#### Clusters 2a and 2b

Clusters 2a and 2b illustrated 13 and 26 proteins after AgNP chronic + acute treatment that decreased and increased, respectively.

In cluster 2a, we found lower amounts of proteins related to cell membrane. This finding supports the evidence indicating that the cell membrane is one of the main targets of the AgNPs. Other membrane-related proteins such as the bacteriophage N4 receptor and the L-arginine ABC transporter-periplasmic binding protein were some of these downregulated proteins. Moreover, to confirm the toxic effect of AgNPs chronic + acute exposure on cell membrane, we observed the downregulation of a phosphate acyltransferase. This protein is involved in fatty acids/phospholipid biosynthesis that possibly contributes to reducing membrane fluidity and stability (Parsons and Rock, 2013).

Bacterial cell wall also seemed to be altered. In AgNP-treated biofilm, we observed the downregulation of the L,D-transpeptidase (LdtD), which is the key enzyme in the peptidoglycan maturation pathway (Vollmer et al., 2008).

The lower adherence of cells on the biofilm and the different distribution patterns within the biofilms observed after 96 h of AgNP exposure (Garuglieri et al., 2018) were also confirmed by proteomic analyses. In fact, chronic and acute AgNP treatments caused the downregulation of some proteins that have a key role in bacterial adhesion, such as flagellins and fimbrial proteins. Flagellum-mediated motility contributes to *E. coli* virulence by enabling the bacterium to escape host immune responses and to colonize new sites within the urinary tract (Lane et al., 2007). *E. coli* fimbrial proteins mediate the binding to receptor structures allowing the bacteria to colonize various host tissues (Krogfelt et al., 1990). The investigated *E. coli* flagellin mutant strains produced less biofilm than the wild-type strain (Zhou et al., 2014).

Beta-glucuronidase was another protein that we found to be downregulated in AgNP-treated samples. β-Glucuronidase and phospholipase are factors in creating colonization surface that facilitate bacteremia and severe infections (Stewart et al., 2007; Biernat et al., 2019).

A transcriptional repressor of the proline utilization regulon (*put*), the PutA protein, belonged to cluster 2a. Proline metabolism strongly influences the oxidative stress tolerance in *E. coli.* As a consequence, *putA* mutant strains are significantly more sensitive to oxidative stress than the wild-type strains, and the overexpression of *putA* restores the oxidative stress resistance (Zhang et al., 2015). Thus, the decrease in the protein PutA under chronic + acute treatments might expose the biofilm to oxidative stress. Garuglieri et al. (2018) observed increased ROS levels at 96 h in anaerobic biofilms subjected to both AgNP acute and chronic treatments at 82 and 55% higher than the control, respectively. However, ROS increase under the acute treatment was more evident than the chronic one. Chen et al. (2017) reported that, under anaerobic conditions, there is a lower production of ROS, which did not affect *Pseudomonas aeruginosa* viability. However, the lack of deleterious mechanisms induced by ROS could cause the adaptation of bacteria and changes in the microbiomes (Niño-Martínez et al., 2019). The relationships between oxidative stress and biofilm development have been widely discussed by Gambino and Cappitelli (2016).

The potassium efflux system KefA domain protein, which is downregulated in this cluster, has a pivotal role in the survivability of bacteria after toxic electrophile exposure. This protein is part of the bacterial protection system, where the electrophiles are detoxified by modulation of the cellular pH (Ferguson et al., 1993; Ozyamak et al., 2010). Zhang et al. (2014) evaluated the active detachment mechanism in the dispersal of *P. aeruginosa* biofilms caused by glutathione-gated potassium efflux, showing biofilm detachment by the activation of glutathione-gated potassium efflux.

Cluster 2b showed the change in abundance of sugar-transport-related regulator, which indicated the alteration of glucose metabolism as also reported in cluster 1. This regulator coordinates the response to glucose-phosphate stress, a condition that occurs when glucose-6-phosphate is accumulated intracellularly and unbroken through the glycolytic pathway (Vanderpool and Gottesman, 2007).

Among upregulated proteins belonging to cluster 2b, we found the disulfide bond reductase, the triosephosphate isomerase, and the uridine-5′-triphosphate-glucose-1-phosphate uridylyltransferase (*UTP*-glucose-1-phosphate uridylyltrans ferase). The latter protein catalyzes the formation of uridine diphosphate-glucose (UDP-glucose) from glucose-1-phosphate and UTP, and it is also required for biofilm formation (Lazarevic et al., 2005). In biofilm and planktonic cultures of *Staphylococcus aureus*, the triosephosphate isomerases were differentially expressed as confirmed by Northern blot analysis (Becker et al., 2001). The altered disulfide bond reductase seems to be involved in the reduction in disulfide bonds between proteins and small molecules (Mac et al., 2008). The disulfide bond formation is also required for biofilm formation and pilus assembly as observed in *Actinomyces oris* biofilms (Lee and Davey, 2017).

#### Clusters 3a and 3b

The clusters 3a and 3b showed eight proteins that were up- and downregulated by the chronic treatments, respectively.

Garuglieri et al. (2018) showed that biofilm grown in the presence of AgNPs for 96 h resulted in a 42.8 ± 11.1% decrease in thickness. Proteomic analysis confirmed this result, and in fact, we observed that, after 96 h of AgNP exposure, different proteins involved in biofilm growth were downregulated (cluster 3a). One of these proteins was the cyclic di-guanylate monophosphate (c-di-GMP) phosphodiesterase (YhjK), which is one of the proteins involved in bacteria motility and biofilm formation (Moreira et al., 2017). Bacteria modulates the intracellular levels of the second messenger c-di-GMP levels to regulate the transition between motile and sessile states. In response to extracellular signals, YhjK is able to modulate the levels of c-di-GMP, mediating the conversion from motile to sessile states (Spurbeck et al., 2012).

In cluster 3a, we also found the involvement of the cell division activator in the reactivation of the DnaA protein, which is one of the main factors controlling the initiation of chromosomal DNA replication (Abe et al., 2016).

The regulator of biofilm through signal secretion protein (BssR), belonging to the upregulated proteins (cluster 3b), is also involved in biofilm formation in *E. coli*. This protein reduces bacterial biofilm by indole regulation (Sutrina et al., 2015).

The formamidopyrimidine-DNA glycosylase also belonged to cluster 3b. This molecule is a DNA repair enzyme that efficiently repairs purines- and pyrimidine-derived lesions induced by hydroxyl radical in DNA (Schalow et al., 2011), suggesting DNA damages due to AgNP chronic exposure.

#### Clusters 4a and 4b

The clusters 4a and 4b showed eight and six proteins that were up- and downregulated by the acute AgNP treatments, respectively.

The primosomal protein, one of the several cell surface proteins required for a stable DNA replication (Zhao et al., 2015), is downregulated only by the acute treatment (cluster 4a).

Another protein downregulated in our analysis was the flagellar brake protein YcgR. This protein is involved in biofilm formation, in particular motility inhibition (Simm et al., 2004; Ryjenkov et al., 2006).

We also found lower amount of the PgaA, which is a tetratricopeptide-repeat-containing outer membrane protein. PgaA forms secretion pores in the outer membrane and translocates the dPNAG polymer from the periplasm to the cell surface. Tetratricopeptide repeat domain of PgaA binds directly to the *N*-deacetylase PgaB and affects critically biofilm formation (Wang et al., 2004; Itoh et al., 2008).

Among the upregulated proteins in cluster 4b, we found the permease and dipeptide transport system permease protein DppB, also known as dipeptide ABC transporter. Zhu et al. (2008) showed that the inactivation of *lm.G_1771* gene, which encodes for a putative ABC transporter permease, increased *Listeria monocytogenes* biofilm.

Sensor kinase protein (RcsC) was also upregulated. It plays an important role in biofilm formation by remodeling the bacterial surface during growth on a solid surface (Ferrières and Clarke, 2003).

We also observed an increase in the amount of the elongation factor Tu, a prokaryotic elongation factor responsible for catalyzing the binding of an aminoacyl-tRNA to the ribosome (Granato et al., 2004; Amimanan et al., 2017). Svensäter et al. (2001) showed that the elongation factor Tu is highly expressed in *Streptococcus mutans* biofilms.

### TEM Analysis of *E. coli* Biofilm Under AgNP Treatments

**FIGURE 5:**
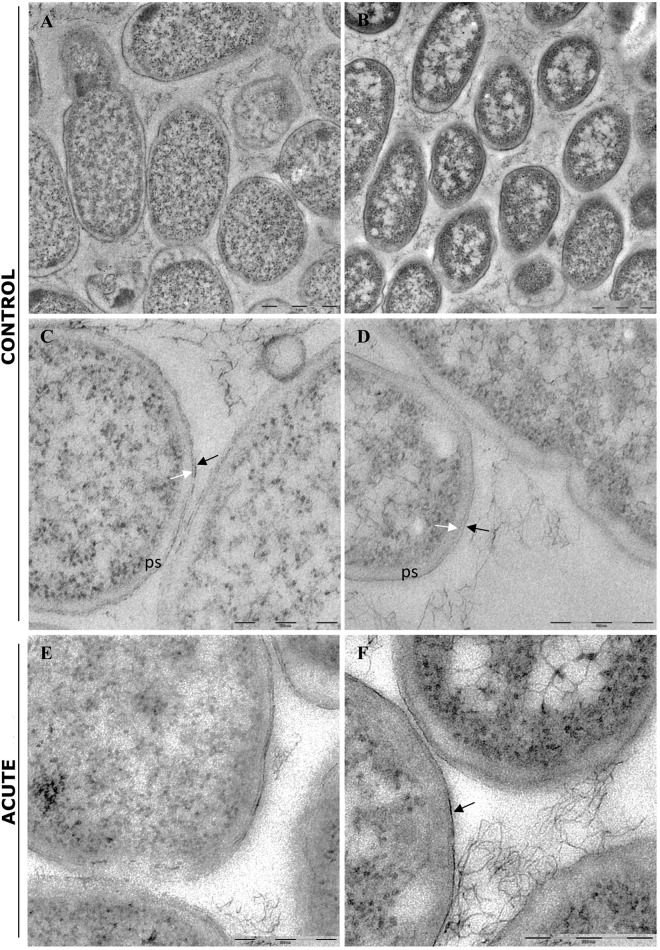
Transmission electron microscopy (TEM) analysis of untreated biofilm (control) and biofilm treated with AgNPs for 24 h (acute treatment). **(A)** Low magnification of inner biofilm showing a uniform granular cytoplasm. **(B)** Low magnification of outer biofilm showing granular cytoplasm surrounding nucleoid. **(C,D)** Inner and outer biofilm showing a typical Gram-negative cell walls, respectively. ps, periplasmic space; black arrow: outer membrane; white arrow: peptidoglycan layers. **(E,F)** Biofilm after AgNP acute treatment. Inner biofilm appeared similar to control **(E)**, while outer biofilm showed a more electrondense outer membrane (arrow). The peptidoglycan layers were less evident. Magnification bars: **(A,B)** 1 μm; **(C–F)** 500 nm.

**FIGURE 6:**
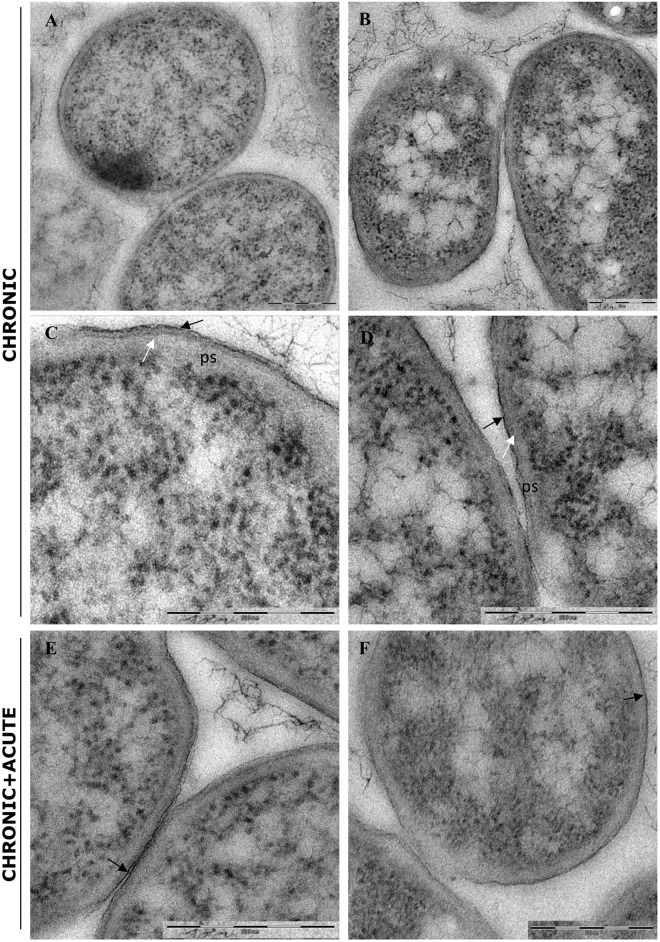
Transmission electron microscopy (TEM) analysis of biofilm grown in the presence of AgNPs for 96 h (chronic treatment) and biofilm grown in the presence of AgNPs for 96 h and treated again for 24 h with AgNPs (chronic + acute treatment). **(A,B)** Inner and outer biofilm showing a modified cell wall with a more electrondense outer membrane (black arrows) and a disorganized peptidoglycan layers (white arrows), respectively. **(C,D)** Biofilm chronic + acute AgNP treatment. No differences are observed compared to biofilm grown in the presence of AgNPs for 96 h (chronic treatment) (outer membrane: black arrows). Magnification bars: **(A–F)** 500 nm.

**FIGURE 7:**
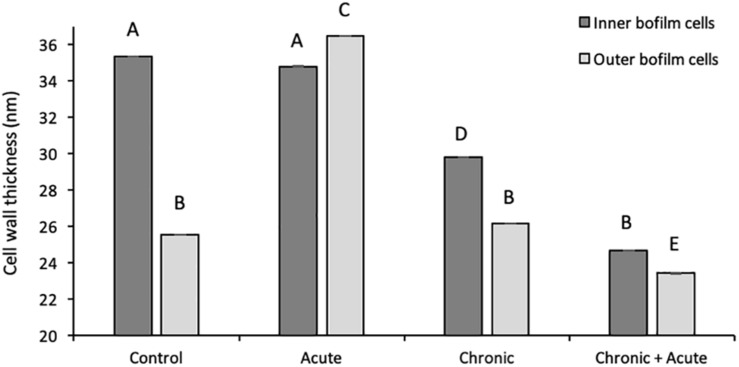
Wall thickness of inner and outer biofilm cells under different treatments. Data represent the mean of independent measurements. Different superscript letters indicate statistically significant differences between conditions.

In control samples, TEM analyses showed that the bacterial cells forming the biofilm have different morphologies. Cells near the polycarbonate membrane (inner biofilm, Figure 5A) showed uniform granular cytoplasm (Rowlett et al., 2017). Cell walls had a typical *E. coli* wall structure, where the periplasmic space (PS) is sandwiched between the plasma membrane and the outer membrane (OM), while the peptidoglycan layer is localized under the OM (Figure 5C) (Beveridge, 1999; Silhavy et al., 2010). Farther away from the polycarbonate membrane (outer biofilm, Figures 5B,D), biofilm cells revealed a more heterogeneous cytoplasm with a granular region surrounding the nucleoid clear area (Figures 5B,D,F), as in the growing cells observed by Rowlett et al. (2017). After AgNP treatment, cytoplasm morphology did not change both in the inner and the outer biofilm layers. However, in the outer biofilm, cell wall OM appeared to be more electron dense than the control (Figure 5F, arrow), suggesting AgNP deposition. Cell wall of the inner biofilm, on the contrary, was similar to the control (compare Figures 5C,E). The presence of an electron-dense OM was also evident in samples exposed to chronic and chronic + acute treatments (Figure 6). The cell wall thickness under all treatments is reported in Figure 7.

## Conclusion

In this study, we demonstrated that AgNPs led to changes in protein expression that is directly related to biofilm formation and maturation. In particular, we observed the downregulation of proteins that have key roles in bacterial adhesion, such as flagellins and fimbrial proteins, which are necessary for biofilm formation (Zhou et al., 2014).

The proteomic investigation revealed many altered membrane- and cell-wall-associated proteins. This result was further confirmed by the reduction in the cell wall thickness, as observed in TEM analyses. The presence of AgNP aggregates in the cytoplasm was not detected in TEM analyses.

Glucose utilization pathways were also altered, and some evidence indicated the possibility of DNA damages, as shown by the upregulation of proteins related to the DNA repair system.

Furthermore, to deepen the understanding of AgNP effects on the gut interactive ecosystem, the proteomic response of Caco-2 intestinal cells to AgNP sublethal concentrations will be carried out.

## Data Availability Statement

The mass spectrometry proteomics data have been deposited in the ProteomeXchange Consortium via the PRIDE (Perez-Riverol et al., 2019) partner repository under the dataset identifier PXD014096.

## Author Contributions

GD, FV, CV, EG, and EO conceived, designed the research, and wrote the manuscript. GD, EG, and EO conducted the experiments. GD performed data analyses. MB and FC contributed critically to the designed research and drafts. All authors read and approved the manuscript.

## Conflict of Interest

The authors declare that the research was conducted in the absence of any commercial or financial relationships that could be construed as a potential conflict of interest.

## Footnotes

1 http://www.coxdocs.org/doku.php?id=maxquant:start

2 http://www.r-project.org

3 https://bioinfogp.cnb.csic.es/tools/venny/
