# Supplementary material for: Label-Free Proteomic Approach to Study the Non-lethal Effects of Silver Nanoparticles on a Gut Bacterium
Source: Front Microbiol. 2019 Dec 4;10:2709. doi: 10.3389/fmicb.2019.02709 (PMC6906586; doi:10.3389/fmicb.2019.02709)
Supplement: Supplementary file 2 [file Table_2.docx]

Table S2. Proteins of the eight most representative expression clusters with the respective intensity values, localizations and log2 fold changes (log2FC).

| **CLUSTER 1a** | | | | **Average intensity (log2)** | | | | **log2_FC** | | |
| --- | --- | --- | --- | --- | --- | --- | --- | --- | --- | --- |
| **Entry** | **Protein names** | **EcoCyc ID** | **Blast name** | **Control** | **Acute** | **Chronic** | **Chronic+acute** | **Acute vs Control** | **Chronic vs Control** | **Chronic+acute vs Control** |
| N2GWD3 | Putative phospholipid ABC transporter-binding protein mlaB | W8TUH8 | phospholipid ABC transporter | 27.80 | 21.85 | 22.12 | 21.96 | -5.95 | -5.68 | -5.84 |
| Q5MDC1 | Malate dehydrogenase (Fragment) | b3236 | malate dehydrogenase | 29.27 | 22.34 | 22.13 | 22.39 | -6.93 | -7.14 | -6.87 |
| N3MZ53 | Citrate synthase family protein (Fragment) | G6198 | 2-methylcitrate synthase | 25.53 | 22.54 | 22.47 | 22.32 | -2.99 | -3.06 | -3.21 |
| W1EZR8 | Cob(I)alamin adenosyltransferase | W8SSB7 | cobinamide adenosyltransferase | 25.11 | 21.99 | 22.04 | 22.13 | -3.12 | -3.08 | -2.98 |
| W8SUQ2 | Putative dehydrogenase (Xanthine dehydrogenase) | G7486 | xanthine dehydrogenase subunit | 25.68 | 22.26 | 22.36 | 22.15 | -3.42 | -3.32 | -3.53 |
| W8SSB7 | GTP cyclohydrolase II (Swarming motility protein) | EG11579 | conserved protein | 25.09 | 21.77 | 21.88 | 21.88 | -3.32 | -3.21 | -3.21 |
| M9GVG3 | Xylose isomerase | b3565 | xylose isomerase | 25.45 | 22.14 | 22.12 | 22.12 | -3.31 | -3.33 | -3.33 |
| T9H4G6 | Uncharacterized protein | G7122 | putative protein | 25.07 | 21.41 | 22.50 | 22.50 | -3.66 | -2.57 | -2.57 |
| V2QTW7 | Protein YdeJ | EG11645 | conserved protein | 26.82 | 21.75 | 21.76 | 22.72 | -5.06 | -5.06 | -4.10 |
| A0A073UM76 | Deleted. | - | - | 26.56 | 22.35 | 21.88 | 21.70 | -4.20 | -4.68 | -4.86 |
| A0A024L0H3 | UDP-4-amino-4-deoxy-L-arabinose--oxoglutarateamin otransferase | G7166 | UDP-L-Ara4O C-4" transaminase | 26.52 | 22.12 | 22.31 | 21.91 | -4.40 | -4.21 | -4.61 |
| W8TUE3 | DNA-binding transcriptional regulator FrlR | G7727 | predicted DNA-binding transcriptional regulator | 26.33 | 21.98 | 21.94 | 21.87 | -4.35 | -4.39 | -4.46 |
| X7PPK7 | Sugar phosphatase SupH | G6425 | sugar phosphatase | 26.15 | 22.33 | 22.39 | 22.22 | -3.82 | -3.76 | -3.93 |
| W8ZXM7 | Uncharacterized protein | EG10018 | regulator of CsrB and CsrC decay | 25.85 | 23.00 | 21.88 | 22.46 | -2.85 | -3.96 | -3.39 |
| U9ZNT2 | Uncharacterized protein (Fragment) | b0345 | T9A3H1 | 25.47 | 22.90 | 22.33 | 21.79 | -2.57 | -3.14 | -3.68 |
| W8TUH8 | Sulfurtransferase TusD | G7714 | sulfur transfer protein complex. TusD subunit | 25.85 | 22.17 | 22.95 | 22.25 | -3.68 | -2.90 | -3.60 |
| **CLUSTER 1b** | | | | **Average intensity** | | | | **log2_FC** | | |
| **Entry** | **Protein names** | **EcoCyc ID** | **Blast name** | **Control** | **Acute** | **Chronic** | **Chronic+acute** | **Acute vs Control** | **Chronic vs Control** | **Chronic+acute vs Control** |
| V8KI28 | Membrane protein | G7398 | inner membrane thiosulfate sulfurtransferase | 22.59 | 27.60 | 27.49 | 27.82 | 5.01 | 4.90 | 5.24 |
| X7PK56 | Acyl-CoA thioester hydrolase YciA | EG11121 | acyl-CoA thioesterase | 22.64 | 27.38 | 27.45 | 27.46 | 4.74 | 4.81 | 4.82 |
| V0XC26 | Uncharacterized protein | G6865 | conserved protein | 22.12 | 27.76 | 27.56 | 27.71 | 5.63 | 5.44 | 5.58 |
| D8BU89 | Putative Threonine/serine transporter TdcC | b3116 | serine / threonine:H<sup>+</sup> symporter TdcC | 22.03 | 27.31 | 27.17 | 27.67 | 5.28 | 5.14 | 5.64 |
| A0A070SU15 | Aldehyde ferredoxin oxidoreductase. domains 2 & 3 family protein | G6901 | predicted oxidoreductase | 21.63 | 27.41 | 27.04 | 27.15 | 5.79 | 5.41 | 5.52 |
| X7PT09 | Glyoxylase I family protein | G6095 | predicted lyase | 22.05 | 27.59 | 27.49 | 26.71 | 5.54 | 5.44 | 4.66 |
| H4L7R8 | Periplasmic binding family protein | b0592 | ferric enterobactin ABC transporter - periplasmic binding protein | 22.31 | 25.87 | 25.90 | 26.17 | 3.56 | 3.60 | 3.86 |
| X7P1Y8 | 5-formyltetrahydrofolate cyclo-ligase | EG11158 | 5-formyltetrahydrofolate cyclo-ligase | 22.35 | 25.70 | 25.81 | 25.58 | 3.35 | 3.46 | 3.23 |
| X7PP91 | Hydrogenase-1 operon protein hyaE | EG10472 | protein involved in quality control of HyaA | 22.06 | 25.64 | 25.80 | 25.84 | 3.59 | 3.74 | 3.78 |
| W1EXD8 | Uncharacterized protein YhjG | EG12251 | predicted outer membrane biogenesis protein | 21.31 | 25.46 | 25.60 | 25.73 | 4.15 | 4.29 | 4.42 |
| H1BXS7 | Glutaminase | G6810 | glutaminase | 21.87 | 26.11 | 26.74 | 26.74 | 4.24 | 4.87 | 4.87 |
| T9A3H1 | Glyoxalase | EG11383 | isoprenoid biosynthesis protein with amidotransferase-like domain | 21.94 | 26.52 | 26.38 | 26.37 | 4.58 | 4.44 | 4.43 |
| I0VLW5 | Uncharacterized protein | G6947 | conserved inner membrane protein | 22.23 | 26.50 | 26.44 | 26.71 | 4.27 | 4.21 | 4.48 |
| N3EY83 | 1-acyl-sn-glycerol-3-phosphate acyltransferase | b3018 | 1-acylglycerol-3-phosphate | 21.76 | 26.43 | 26.84 | 26.50 | 4.67 | 5.09 | 4.75 |
| **W8TQQ2** | | | | **Average intensity** | | | | **log2_FC** | | |
| **Entry** | **Protein names** | **EcoCyc ID** | **Blast name** | **Control** | **Acute** | **Chronic** | **Chronic+acute** | **Acute vs Control** | **Chronic vs Control** | **Chronic+acute vs Control** |
| V6FL94 | Bifunctional protein PutA | b1014 | fused PutA transcriptional repressor / proline dehydrogenase | 26.80 | 27.14 | 27.29 | 22.51 | 0.34 | 0.50 | -4.28 |
| X7PM64 | Nicotinate-nucleotide--dimethylbenzimidazole phosphoribosyltransferase | b1991 | nicotinate-nucleotide dimethylbenzimidazole phosphoribosyltransferase | 25.63 | 25.69 | 25.57 | 21.94 | 0.06 | -0.06 | -3.69 |
| M8JPP3 | Uncharacterized protein | EG12705 | putative protein | 25.47 | 25.51 | 25.39 | 22.04 | 0.04 | -0.08 | -3.43 |
| H9UVX2 | Formate hydrogenlyase subunit 2 | b2724 | hydrogenase 3. Fe-S subunit | 25.71 | 25.27 | 26.07 | 21.40 | -0.44 | 0.36 | -4.30 |
| V1AK89 | Potassium efflux system KefA domain protein | G7840 | mechanosensitive channel of miniconductance MscM | 26.04 | 26.15 | 26.02 | 22.03 | 0.11 | -0.01 | -4.01 |
| X7NZF9 | Orotate phosphoribosyltransferase (OPRT) | b3642 | orotate phosphoribosyltransferase | 25.57 | 25.50 | 25.41 | 21.69 | -0.07 | -0.16 | -3.88 |
| W1EZC8 | Mhp operon transcriptional activator | G6201 | MhpR transcriptional activator | 25.95 | 25.88 | 25.61 | 21.82 | -0.07 | -0.34 | -4.13 |
| A0A125X4Z9 | Putative L.D-transpeptidase YcbB | b0925 | L.D-transpeptidase LdtD | 26.53 | 26.34 | 26.50 | 22.35 | -0.20 | -0.03 | -4.18 |
| A0A0A0FP09 | Citrate lyase acyl carrier protein (Gamma chain) | b0617 | citrate lyase. acyl carrier &gamma | 26.25 | 26.18 | 26.14 | 22.24 | -0.07 | -0.11 | -4.01 |
| F4TGG1 | Beta-glucuronidase (GUS) | b1617 | β-D-glucuronidase | 26.36 | 26.35 | 26.34 | 22.38 | -0.01 | -0.02 | -3.98 |
| W8T497 | Fructoselysine 3-epimerase (Protein FrlC) | G7724 | fructoselysine 3-epimerase | 26.13 | 25.97 | 26.22 | 22.16 | -0.16 | 0.09 | -3.97 |
| L3QSY0 | Bacteriophage N4 receptor. outer membrane subunit | EG11740 | bacteriophage N4 receptor. outer membrane protein | 25.83 | 26.00 | 25.94 | 21.89 | 0.17 | 0.10 | -3.95 |
| W8T732 | Fructose-6-phosphate aldolase | EG11905 | fructose 6-phosphate aldolase 2 | 26.17 | 26.13 | 26.01 | 21.74 | -0.04 | -0.16 | -4.43 |
| X7P2F2 | Nucleoside triphosphate pyrophosphohydrolase | b2781 | nucleoside triphosphate pyrophosphohydrolase | 26.83 | 26.78 | 26.32 | 22.11 | -0.05 | -0.52 | -4.73 |
| W1HGW9 | Phosphate acyltransferase | b1090 | fatty acid/phospholipid synthesis protein | 27.19 | 26.75 | 26.77 | 21.91 | -0.44 | -0.43 | -5.28 |
| S1HRR2 | Ethanolamine utilization protein EutL | b4320 | ethanolamine utilization microcompartment | 26.62 | 26.70 | 26.55 | 22.57 | 0.09 | -0.07 | -4.04 |
| D7ZQU6 | Fimbrial protein | EG10315 | minor fimbrial subunit. D-mannose specific adhesin | 26.79 | 26.87 | 26.41 | 22.55 | 0.07 | -0.38 | -4.24 |
| E1J0C8 | BolA-like protein (Fragment) | G7657 | predicted DNA-binding transcriptional regulator | 26.63 | 26.82 | 26.84 | 22.23 | 0.19 | 0.20 | -4.40 |
| I0VR27 | Uncharacterized protein | G6178 | putative protein | 26.77 | 26.83 | 26.89 | 22.19 | 0.07 | 0.13 | -4.57 |
| W8TQQ2 | ABC transporter arginine-binding protein 1 | b0860 | L-arginine ABC transporter - periplasmic binding protein | 27.02 | 26.81 | 26.88 | 21.76 | -0.21 | -0.14 | -5.26 |
| X7NN96 | D-aminoacyl-tRNA deacylase | EG11852 | D-Tyr-tRNA<sup>Tyr</sup> deacylase | 27.22 | 27.12 | 27.35 | 22.01 | -0.10 | 0.13 | -5.21 |
| A0A080G0R7 | Flagellin | EG10321 | flagellar biosynthesis | 28.90 | 28.95 | 28.52 | 21.35 | 0.05 | -0.38 | -7.55 |
| W1V135 | Rac prophage repressor | G6680 | Rac prophage | 28.24 | 27.80 | 27.46 | 22.44 | -0.45 | -0.79 | -5.80 |
| X7PSS2 | Protein phnB | EG10712 | conserved protein | 27.84 | 27.86 | 27.89 | 21.61 | 0.02 | 0.06 | -6.23 |
| W1WZP5 | Uncharacterized protein (Fragment) | G6380 | predicted enzyme subunit | 28.32 | 28.15 | 28.26 | 22.38 | -0.17 | -0.06 | -5.94 |
| X7P1P3 | Flavoprotein | G7448 | predicted flavoprotein | 28.54 | 28.36 | 28.48 | 22.86 | -0.18 | -0.06 | -5.67 |
| **CLUSTER 2b** | | | | **Average intensity** | | | | **log2_FC** | | |
| **Entry** | **Protein names** | **EcoCyc ID** | **Blast name** | **Control** | **Acute** | **Chronic** | **Chronic+acute** | **Acute vs Control** | **Chronic vs Control** | **Chronic+acute vs Control** |
| L9HS09 | Homoserine O-succinyltransferase | b4013 | homoserine <i>O</i>-succinyltransferase | 22.03 | 22.09 | 22.21 | 24.25 | 0.06 | 0.18 | 2.22 |
| V1AK56 | HTH-type transcriptional regulator SgrR | EG12094 | SgrR DNA-binding transcriptional dual regulator | 21.55 | 22.06 | 21.25 | 24.01 | 0.51 | -0.29 | 2.47 |
| M8JQE9 | Uncharacterized protein | G7933 | hypothetical protein | 22.48 | 22.55 | 22.08 | 24.86 | 0.08 | -0.40 | 2.38 |
| N4PEF7 | Bacterial transcriptional regulator family protein | G6144 | CP4-6 prophage; predicted DNA-binding transcriptional regulator | 22.42 | 22.06 | 21.56 | 25.01 | -0.36 | -0.86 | 2.59 |
| X7PKB4 | Nucleoside triphosphate hydrolase | G6659 | conserved protein | 22.00 | 22.56 | 22.87 | 25.86 | 0.56 | 0.87 | 3.86 |
| V1AQH4 | Multiple antibiotic resistance protein MarR | b1530 | MarR DNA-binding transcriptional repressor | 21.83 | 22.16 | 22.25 | 26.48 | 0.33 | 0.42 | 4.65 |
| M9G6I6 | PTS system. glucose-like IIB component domain protein | b1621 | MalX PTS permease | 21.92 | 22.09 | 22.21 | 27.04 | 0.17 | 0.29 | 5.12 |
| I0VLY2 | Isochorismatase family protein | b1768 | nicotinamidase / pyrazinamidase | 22.57 | 22.04 | 22.62 | 27.27 | -0.53 | 0.05 | 4.70 |
| A0A075LE10 | Deleted. | - | - | 21.79 | 22.21 | 21.71 | 26.47 | 0.42 | -0.08 | 4.67 |
| A0A0T5XG98 | ATP-dependent RNA helicase HrpA | G6732 | ATP-dependent helicase | 21.58 | 21.53 | 21.15 | 26.35 | -0.05 | -0.43 | 4.77 |
| A0A0T5XIH6 | Triosephosphate isomerase | b3919 | triosephosphate isomerase | 21.78 | 21.93 | 22.42 | 28.27 | 0.15 | 0.64 | 6.50 |
| W1FRC5 | UTP--glucose-1-phosphate uridylyltransferase | G7093 | UTP:glucose-1-phosphate uridylyltransferase | 22.17 | 22.02 | 22.41 | 29.43 | -0.14 | 0.24 | 7.26 |
| H4HYU4 | Disulfide bond reductase | G7194 | disulfide reductase | 22.18 | 21.67 | 22.46 | 29.82 | -0.51 | 0.28 | 7.64 |
| **CLUSTER 3a** | | | | **Average intensity** | | | | **log2_FC** | | |
| **Entry** | **Protein names** | **EcoCyc ID** | **Blast name** | **Control** | **Acute** | **Chronic** | **Chronic+acute** | **Acute vs Control** | **Chronic vs Control** | **Chronic+acute vs Control** |
| W1HH94 | Hypothetical lipoprotein YajG | EG12182 | predicted lipoprotein | 28.92 | 29.15 | 22.20 | 21.98 | 0.23 | -6.72 | -6.94 |
| H4IYF9 | Acid-resistance membrane protein (Fragment) | EG11495 | acid-resistance membrane protein | 29.40 | 29.32 | 21.47 | 21.92 | -0.07 | -7.92 | -7.48 |
| E6BUH9 | Uncharacterized protein (Fragment) | G6818 | Qin prophage; cold shock gene. predicted DNA-binding transcriptional | 26.31 | 26.10 | 21.76 | 22.54 | -0.21 | -4.55 | -3.76 |
| H9UY84 | Protein yhjK | EG12256 | predicted c-di-GMP phosphodiesterase | 26.09 | 25.84 | 22.27 | 22.68 | -0.26 | -3.83 | -3.41 |
| W1WIP3 | Uncharacterized protein (Fragment) | G6963 | predicted oxidoreductase | 26.42 | 26.11 | 22.69 | 22.63 | -0.31 | -3.73 | -3.79 |
| U9YIM4 | Lipopolysaccharide heptosyltransferase I | EG11189 | ADP-heptose:LPS heptosyltransferase I | 25.94 | 25.90 | 22.94 | 22.52 | -0.04 | -3.00 | -3.42 |
| N3KCB2 | ADP compounds hydrolase nudE | G7740 | ADP-sugar pyrophosphorylase | 26.96 | 26.39 | 22.37 | 22.78 | -0.57 | -4.59 | -4.18 |
| W8TE37 | Cell division activator CedA | G6936 | cell division modulator | 25.15 | 25.38 | 22.09 | 21.90 | 0.24 | -3.05 | -3.25 |
| **CLUSTER 3b** | | | | **Average intensity** | | | | **log2_FC** | | |
| **Entry** | **Protein names** | **EcoCyc ID** | **Blast name** | **Control** | **Acute** | **Chronic** | **Chronic+acute** | **Acute vs Control** | **Chronic vs Control** | **Chronic+acute vs Control** |
| X7NZ47 | Formamidopyrimidine-DNA glycosylase | EG10329 | formamidopyrimidine DNA glycosylase | 21.16 | 21.99 | 26.49 | 26.51 | 0.83 | 5.33 | 5.35 |
| J7R3E7 | NADH-quinone oxidoreductase subunit N | b2276 | NADH:ubiquinone oxidoreductase. membrane subunit N | 21.91 | 22.14 | 27.61 | 27.92 | 0.23 | 5.70 | 6.01 |
| X7P2A1 | 5'/3'-nucleotidase SurE | EG11817 | broad specificity 5'(3')-nucleotidase and polyphosphatase | 21.92 | 22.43 | 27.59 | 27.36 | 0.52 | 5.67 | 5.44 |
| V0ZW43 | Uncharacterized protein | G6978 | conserved protein | 22.48 | 22.19 | 27.10 | 27.15 | -0.29 | 4.62 | 4.67 |
| W8ZZG5 | Uncharacterized protein | EG11201 | conserved protein | 22.43 | 22.70 | 26.83 | 27.01 | 0.27 | 4.41 | 4.58 |
| W1EUF9 | Biofilm regulator BssR | G6436 | regulator of biofilm formation | 21.78 | 22.11 | 29.58 | 28.23 | 0.33 | 7.80 | 6.46 |
| A0A073H4V0 | Aerobic C4-dicarboxylate transport protein | b3528 | C4 dicarboxylate / orotate:H<sup>+</sup> symporter | 21.74 | 22.03 | 29.40 | 28.76 | 0.29 | 7.66 | 7.02 |
| M8LEN8 | Glutamate decarboxylase | b1493 | glutamate decarboxylase B subunit | 22.23 | 22.10 | 29.17 | 29.02 | -0.13 | 6.94 | 6.79 |
| **CLUSTER 4a** | | | | **Average intensity** | | | | **log2_FC** | | |
| **Entry** | **Protein names** | **EcoCyc ID** | **Blast name** | **Control** | **Acute** | **Chronic** | **Chronic+acute** | **Acute vs Control** | **Chronic vs Control** | **Chronic+acute vs Control** |
| V0AGY1 | Flagellar brake protein YcgR | G6623 | molecular brake that regulates flagellar motility in response | 25.03 | 22.52 | 25.50 | 22.13 | -2.51 | 0.47 | -2.90 |
| V0VQP7 | Repressor of phase-1 flagellin | b2935 | transketolase I | 25.11 | 22.39 | 25.29 | 21.93 | -2.72 | 0.18 | -3.18 |
| V1AI75 | Octanoyltransferase | EG11591 | lipoyl(octanoyl) transferase | 26.88 | 21.82 | 26.82 | 22.75 | -5.06 | -0.06 | -4.14 |
| A0A0G3J5W9 | Uncharacterized protein | G7461 | putative protein | 27.24 | 22.71 | 26.84 | 22.10 | -4.54 | -0.41 | -5.14 |
| X7PUN5 | Primosomal protein 1 | EG10244 | primosomal protein DnaT | 25.93 | 21.93 | 25.85 | 22.27 | -4.00 | -0.08 | -3.66 |
| W8ZM82 | SsnA protein | G7498 | predicted chlorohydrolase/aminohydrolase | 26.31 | 21.82 | 26.27 | 21.71 | -4.49 | -0.05 | -4.60 |
| A0A125XBS5 | L-threonine dehydratase | b3772 | threonine deaminase | 26.36 | 22.29 | 26.13 | 22.22 | -4.06 | -0.22 | -4.14 |
| V1ALG4 | TPR repeat region | G7510 | FAD assembly factor | 27.52 | 21.74 | 27.27 | 22.51 | -5.78 | -0.25 | -5.01 |
| **CLUSTER 4b** | | | | **Average intensity** | | | | **log2_FC** | | |
| **Entry** | **Protein names** | **EcoCyc ID** | **Blast name** | **Control** | **Acute** | **Chronic** | **Chronic+acute** | **Acute vs Control** | **Chronic vs Control** | **Chronic+acute vs Control** |
| A0A070SLJ2 | Permease | b0336 | cytosine transporter | 22.37 | 25.77 | 22.16 | 26.10 | 3.41 | -0.21 | 3.73 |
| W1XD85 | Dipeptide transport system permease protein dppB (Fragment) | b3543 | dipeptide ABC transporter - predicted membrane subunit | 22.65 | 25.76 | 21.73 | 25.64 | 3.10 | -0.93 | 2.98 |
| V8FMC9 | RNA helicase | EG12329 | putative ATP-dependent RNA helicase HrpB | 22.38 | 25.77 | 22.25 | 25.91 | 3.39 | -0.13 | 3.53 |
| N2GVK9 | HTH-type transcriptional regulator ulaR | G7854 | UlaR DNA-binding transcriptional repressor | 22.02 | 26.48 | 21.78 | 26.42 | 4.46 | -0.24 | 4.41 |
| F4SG61 | Sensor kinase protein RcsC (Capsular synthesis regulatorcomponent C) | b2218 | RcsC sensory histidine kinase | 22.18 | 25.52 | 22.38 | 25.88 | 3.34 | 0.21 | 3.70 |
| A0A0F3TYE0 | Elongation factor Tu ) | EG11037 | elongation factor Tu | 22.76 | 26.12 | 22.29 | 27.66 | 3.36 | -0.47 | 4.90 |
